# Reading and Writing Profiles in Twice-Exceptional Adolescents with Intellectual Giftedness and Dyslexia

**DOI:** 10.3390/jintelligence14060108

**Published:** 2026-06-12

**Authors:** Samuel Alonso Benito, Luz Florinda Pérez Sánchez, Ángeles Bueno Villaverde

**Affiliations:** Department of Education, Faculty of Education, Universidad Camilo Jose Cela, 28692 Madrid, Spain

**Keywords:** intellectual giftedness, dyslexia, twice-exceptionality, adolescents, learning disorder, reading processes, writing processes

## Abstract

This study provides empirical evidence on the reading and writing profile of adolescents with twice-exceptionality (2e), specifically those presenting both intellectual giftedness and dyslexia (G-D). Using a quantitative approach, the performance of the G-D group was compared with that of gifted students without dyslexia (G) and students with dyslexia without intellectual giftedness (D) in a sample of Spanish secondary school students. The results indicate that G-D adolescents exhibit a heterogeneous performance pattern across literacy-related measures, characterized by relative weaknesses in lexical and syntactic processes alongside comparatively stronger performance in semantic skills. Although they do not systematically outperform or fall behind the other groups, in specific subtests, G-D students show significantly higher scores than the D group and lower scores than the G group in global reading measures, particularly in the General Reading Index and comprehension tasks. These findings highlight the heterogeneous nature of the G-D profile and suggest that cognitive strengths associated with intellectual giftedness may partially compensate for difficulties related to dyslexia. Understanding this dual profile is essential for improving identification processes and for designing more precise and responsive interventions.

## 1. Introduction

Twice exceptionality (2e), defined as the co-occurrence of intellectual giftedness and a learning disorder, represents a complex cognitive profile characterized by the interaction of strengths and difficulties across multiple domains. In the case of giftedness and dyslexia, this dual condition poses a particular challenge for research, as cognitive strengths may mask underlying difficulties, while deficits may obscure high ability (Assouline et al., 2010; Maddocks, 2018; Nicpon et al., 2011). Understanding how these interacting factors influence adolescents requires moving beyond academic performance to examine the underlying processes involved. From this perspective, twice exceptionality can be conceptualized as a heterogeneous profile in which different cognitive mechanisms contribute to this complexity, making its precise characterization essential for accurate identification, understanding, and the development of tailored responses to their specific needs.

Recent transdiagnostic approaches to neurodevelopmental disorders provide a useful framework for interpreting twice-exceptionality. Astle et al. (2022) argue that an overreliance on rigid diagnostic categories may limit the understanding of heterogeneous neurodevelopmental profiles, particularly when individual needs, within-category variability, and overlap across conditions are not adequately captured. From this perspective, twice-exceptionality should be understood not only as the categorical co-occurrence of giftedness and dyslexia but also as a complex profile in which cognitive strengths and learning-related vulnerabilities interact. This approach is especially relevant for G-D students, whose literacy performance may reflect both risk factors associated with dyslexia and protective or compensatory factors associated with high cognitive ability. Therefore, adopting a transdiagnostic lens may contribute to a more nuanced interpretation of the heterogeneous reading and writing profiles observed in this population.

Giftedness is currently understood as a complex construct whose conceptualization has evolved toward multidimensional approaches. In this regard, Sternberg’s triarchic theory (Sternberg, 1985) conceptualizes giftedness as the integration of analytical, creative, and practical intelligences. Subsequently, Gagné’s Differentiated Model of Giftedness and Talent (Gagné, 2004) distinguishes between natural abilities (gifts) and systematically developed skills (talents), emphasizing the role of personal and environmental factors. Finally, Renzulli’s three-ring model (Renzulli, 2011) proposes that giftedness emerges from the interaction between above-average ability, creativity, and task commitment. However, in practice, identification continues to rely primarily on cognitive assessments, such as intelligence quotient (IQ) tests (Hodges et al., 2018; Kuznetsova et al., 2024; Silverman, 2018). Within this framework, the most widely used criterion is an IQ score of 130 or above, corresponding to approximately 2.28% of the population (François-Sévigny et al., 2025; Tordjman & Kermarrec, 2019). Nevertheless, giftedness does not necessarily guarantee high academic achievement, as a significant proportion of students may experience underachievement, with estimates reaching up to 50% in some cases (Baudry et al., 2026). This discrepancy between cognitive potential and academic performance may be explained by the interaction of several personal and contextual factors, including motivational difficulties, self-efficacy, goal valuation, perceptions of environmental support, self-regulation, and the quality of teacher–student and family relationships (Barbier et al., 2019; Baudry et al., 2026). Moreover, these difficulties often become more apparent at later educational stages, such as secondary education, when academic demands increase and students are expected to use more autonomous and self-regulated learning strategies (Barbier et al., 2019). These findings highlight the influence of personal and contextual factors and underscore the need for early identification and the implementation of differentiated educational strategies (Cross & Coleman, 2014; National Association for Gifted Children, 2019; Pérez & Freitas, 2016).

One of the most common learning disorders is dyslexia, classified in the DSM-5-TR as a neurodevelopmental disorder that affects between 5% and 13% of the population (American Psychiatric Association, 2022; Krafnick & Evans, 2019). Its diagnosis requires clinical judgment and is not dependent on intellectual ability, as it can occur in individuals with high, average, or low intelligence. Dyslexia is characterized by significant difficulties in reading and spelling at the word level, particularly in decoding and spelling fluency (Snowling et al., 2020). Its origin is linked to alterations in phonological processing, affecting sequencing, auditory discrimination, and the mapping between sounds and visual symbols, which are often present from preschool and interfere with the relationship between orthography and phonology, vocabulary acquisition, short-term verbal memory, and word retrieval (Snowling et al., 2019; Vellutino et al., 2004). Moreover, some individuals with dyslexia present difficulties beyond the phonological domain, such as impairments in encoding letter positions, which lead to errors in words that require precise letter order (e.g., pirates vs. parties; smile vs. slime) (Kohnen et al., 2012). During adolescence, these difficulties may become more pronounced as increasing academic demands place greater pressure on reading and writing skills. As a result, students with dyslexia are at greater risk of poor academic performance and of experiencing high levels of stress and frustration (Goldston et al., 2007). Longitudinal studies indicate that these literacy difficulties may persist into adulthood and are associated with lower educational and occupational achievement, potentially leading to frustration, reduced self-esteem, and lower motivation toward reading and writing tasks (Eissa, 2010; Maughan et al., 2020).

Twice exceptionality, defined as the co-occurrence of intellectual giftedness and dyslexia, remains under-researched despite its importance for adequate identification and support. The coexistence of both conditions can lead to mutual masking, complicating diagnosis and intervention, while prevalence estimates remain uncertain due to these challenges and variability in diagnostic criteria (Assouline et al., 2010; Kranz et al., 2024; Maddocks, 2018). As a result, these students face complex and interacting challenges. Intellectual giftedness in adolescence is associated with both high potential and vulnerability, as individuals often lack the opportunities and resources necessary to fully develop their abilities and face additional social and emotional challenges (Moon & Dixon, 2021). At the same time, dyslexia negatively affects academic performance, well-being, and self-esteem, contributing to anxiety, depression, and social difficulties (Carroll & Iles, 2006; Knivsberg & Andreassen, 2008; Maughan et al., 2003). The absence of appropriate support can intensify these effects, leading students to feel misunderstood and different from their peers, which further hinders their integration both in school and in social contexts (Daniel et al., 2006; Eissa, 2010).

Although research on G-D has advanced, significant challenges remain. Studies such as that of Berninger and Abbott (2013) have explored this relationship using a verbal-reasoning-based IQ score of 120 to identify giftedness. Their findings indicate that children with superior verbal reasoning demonstrate strengths in morphological, phonological, and orthographic encoding, as well as in the phonological loop, orally mediated syntactic encoding, and word-level reading and spelling skills. However, they also show difficulties in orthographic loops and in supervisory attention related to inhibition and self-control. Similarly, studies by van Viersen et al. (2015, 2016) indicate that G-D students perform well in verbal and visuospatial working memory, grammar, vocabulary, and short-term verbal memory but perform worse in phoneme deletion and transposition, reading, spelling, and rapid automatized naming. This pattern is consistent with recent findings highlighting that G-D students exhibit a mixed cognitive and literacy profile, in which strengths in higher-order language and reasoning abilities may partially compensate for persistent phonological deficits, resulting in heterogeneous performance across literacy tasks (Alonso Benito et al., 2026). These findings suggest that G-D students may show relative strengths in semantic and syntactic abilities, together with preserved verbal and visuospatial working memory skills, understood as the ability to temporarily store and manipulate information during complex cognitive tasks. These strengths, reported in previous studies, may partially compensate for the deficits associated with dyslexia. Overall, these findings indicate that the profile of gifted students with dyslexia is not solely defined by deficits but by the interaction between cognitive strengths and weaknesses, which may give rise to compensatory processes.

Despite advances in research on 2e in children, knowledge about this condition in adolescents remains limited. In this context, one of the few relevant studies is that of van Viersen et al. (2019), who examined gifted students aged 12–14 previously diagnosed with dyslexia. The study assessed reading and spelling skills to determine whether literacy difficulties had persisted or resolved. Comparisons between gifted students with persistent and resolved dyslexia showed that those with resolved difficulties demonstrated stronger linguistic abilities, including verbal working memory, grammar, and vocabulary. However, even this group continued to exhibit deficits in phonological awareness and impairments in rapid automatized naming. The findings suggest that, although some underlying deficits may improve over time, this is not the case for all individuals.

Nevertheless, there is still a lack of systematic evidence clearly characterizing the reading and writing profiles of G-D adolescents, particularly in studies that directly compare this group with both gifted- and dyslexic-only peers using comparable measures. This limitation makes it difficult to disentangle their relative strengths and weaknesses. In addition, differences in selection methods, identification criteria, assessed skills, and measurement tools further hinder comparisons across studies (Kranz et al., 2024). Many of these adolescents remain unidentified, as they often do not stand out either positively or negatively. Although they do not always exhibit severe literacy difficulties since their reading and writing problems are often milder or partially compensated, this does not eliminate their vulnerability to academic failure (Miles et al., 2003; Nielsen, 2002; Snowling & Hulme, 2012; van Viersen et al., 2019). In light of these limitations, the present study aims to provide a more detailed understanding of the reading and writing profile of G-D adolescents.

In this context, the present study focuses on reading and writing processes because they constitute a core area of difficulty in dyslexia and are central to academic functioning during secondary education. This focus is especially relevant in G-D adolescents, as previous research suggests that their high cognitive or language-related strengths may coexist with persistent difficulties in lower-level literacy processes, such as decoding, spelling, reading fluency, and phonological processing (van Viersen et al., 2019; van Viersen et al., 2015; van Viersen et al., 2016). Moreover, adolescence represents a particularly important stage for examining these profiles, since secondary education involves increasing academic demands, more complex written materials, and greater expectations for autonomy and self-regulated learning, which may make previously compensated difficulties more visible (Barbier et al., 2019). Therefore, examining the reading and writing profile of these adolescents may contribute to a clearer understanding of how cognitive strengths and literacy-related difficulties interact to shape their academic functioning. The specific aims of this study are:

1. To characterize the reading and writing profile of G-D adolescents across different literacy-related measures.
2. To compare the performance of G-D adolescents with that of gifted students without dyslexia (G) and students with dyslexia without intellectual giftedness (D).
3. To identify the strengths and weaknesses of G-D adolescents across lexical, syntactic, and semantic domains.

Based on previous evidence suggesting that dyslexia is primarily associated with difficulties in lower-level literacy processes, whereas giftedness may support higher-order language and comprehension skills, it is hypothesized that G-D adolescents will show lower performance in tasks involving lexical and syntactic skills, while performing relatively better in tasks involving semantic processing. The findings of this study are expected to contribute to a more precise characterization of this population and to inform the development of more targeted and effective educational interventions.

## 2. Materials and Methods

### 2.1. Participants

Assessments were conducted individually between 2017 and 2025 at the Huerta del Rey Psychological and Educational Center (Valladolid, Spain), a specialized facility with an environment specifically adapted for this purpose. Participants were enrolled in 1st to 4th years of secondary education and 1st and 2nd years of high school, were native Spanish speakers, came from different regions of Spain, and had predominantly middle-to-high socioeconomic backgrounds. In all cases, prior informed consent was obtained from the families.

Participants were classified as students with intellectual giftedness based on their performance on the General Ability Index (GAI) of the Wechsler Intelligence Scale for Children–Fifth Edition (Wechsler, 2014), with a cutoff score of 130 or above. Research indicates that the GAI may offer a more accurate estimate of cognitive ability in twice-exceptional populations, as these individuals often exhibit strong verbal comprehension and fluid reasoning skills, while relative weaknesses in processing speed and working memory may artificially lower their Full Scale IQ (FSIQ). Because the GAI does not include the latter components, it is considered a more representative indicator of their cognitive potential in such cases (Assouline et al., 2010; Maddocks, 2020; Toffalini et al., 2017). This criterion is also consistent with a transdiagnostic interpretation of twice-exceptionality, as it allows the identification of high cognitive potential while acknowledging that specific cognitive or academic difficulties may coexist within the same individual (Astle et al., 2022). In this sense, the use of the GAI is particularly relevant for examining G-D profiles, where strengths associated with intellectual giftedness may interact with learning-related vulnerabilities associated with dyslexia.

Participants in the dyslexic groups had an established diagnosis of dyslexia in the clinical records of the center. All diagnoses were made by the same professional, a PhD in psychology specializing in child and adolescent neuropsychology, who was fully accredited and had extensive experience in this field. In the present retrospective study, this diagnosis was used as a grouping criterion, while the analyses focused on characterizing reading and writing performance across the three study groups.

After applying the inclusion criteria, the final sample consisted of 111 students, divided into three groups: students with intellectual giftedness without dyslexia (G; *n* = 46), students with intellectual giftedness and dyslexia (G-D; *n* = 33), and students with dyslexia without intellectual giftedness (D; *n* = 32).

### 2.2. Instruments

Wechsler Intelligence Scale for Children–Fifth Edition (WISC-V; Wechsler, 2014):

- Intellectual level: The General Ability Index (GAI) was computed from subtest scores and provides an assessment of intellectual ability while attenuating the influence of working memory and processing speed. This index is particularly appropriate for the assessment of individuals with learning disorders. The reliability coefficient reported in the manual is above 0.92.
- Processing speed: This primary index assesses the efficiency and rapidity with which individuals process simple visual information, allocate selective attention, and discriminate relevant stimuli. It reflects mental agility and the capacity to process information quickly and accurately. This index was included because processing speed may be particularly relevant for understanding performance in reading and writing tasks, especially when these require rapid and accurate processing of information. The reliability coefficient reported in the manual is 0.88.

PROESC: Writing Processes Assessment Battery (Cuetos et al., 2004). The reliability coefficient reported in the manual for all tests is 0.82:

- Syllable dictation: This task consists of the dictation of 25 syllables representing different syllabic structures. It assesses the student’s ability to transcribe orally presented syllables. Responses are recorded in written form and analyzed to evaluate phonological processing and accuracy in grapheme–phoneme correspondence.
- Word dictation: This task consists of the dictation of two lists, each containing 25 words. First, List A is dictated, composed of words with arbitrary spelling; the score reflects performance in this category. Examples of words from this list include “jefe, zanahoria, lluvia, and ahorro”. Then, List B is dictated, which includes words that follow spelling rules and is used to calculate the rule-based spelling score. Examples of words from this list include “cantaba, tiempo, buey, and escribir”.
- Pseudoword dictation: This task involves the dictation of 25 invented (nonsense) words that participants must write correctly. A total score is obtained from the full list, while the last 15 words, which follow spelling rules, are used to calculate a specific score in this category.
- Story writing: This task assesses writing ability through the creation of a short story. Students may choose any story, whether well-known or less popular, as long as it fits within a designated space and includes a title. Evaluation focuses on content, coherence, and style.
- Composition writing: This task requires the student to produce a written composition on an animal of their choice. The text must be coherent, complete, and fit within the allocated space. Evaluation focuses on content quality and written presentation.

PROLEC-SE-R. Reading Processes Assessment Battery for Secondary and High School Students (Cuetos et al., 2016):

Main Scores: These reflect performance and achievement on the tests used to evaluate each process.

- Lexical Selection: The participant must classify 50 words, distinguishing between 25 real and 25 invented words. The pseudowords are formed from real roots and suffixes, creating fictitious terms. This assessment measures accuracy and speed in word recognition without needing to access their meaning. The reliability coefficient reported in the manual is 0.86.
- Semantic Categorization: The task requires classifying 90 words, identifying which are animal names and which are not. This test measures the speed of access to word meaning, as it requires not only recognition but also comprehension. The reliability coefficient reported in the manual is 0.97.
- Word Reading: The student must read aloud four lists of 24 words each, categorized by length and frequency. Errors and reading time are recorded to assess the ability to retrieve pronunciation from spelling. Performance differences based on frequency and length indicate the use of lexical and sublexical pathways. A greater length effect suggests sublexical use, typical of less skilled readers, while a reduced effect indicates more efficient lexical use, characteristic of expert readers. The reliability coefficient reported in the manual is 0.95.
- Pseudoword Reading: The student reads aloud two lists of pseudowords, one with short terms and another with long ones, with errors and reading time recorded. Since pseudowords have no meaning, the test evaluates the use of the sublexical route, i.e., the ability to convert letters into sounds. The reliability coefficient reported in the manual is 0.77.
- Grammatical Structures I: The student must select the sentence that best describes each of the 24 presented pictures. Each picture is accompanied by three options, including six types of different grammatical structures. It assesses the ability to process grammatically complex sentences, correctly assigning syntactic roles to each element. The reliability coefficient reported in the manual is 0.81.
- Grammaticality Judgments: This task involves evaluating 35 sentences to determine which are grammatically correct and which are incorrect. It is a speed task, as it must be completed within two minutes. The evaluation measures the ability to syntactically process sentences, identifying those with grammatical anomalies. The reliability coefficient reported in the manual is 0.89.
- Grammatical Structures II: This assesses the ability to process sentences with different syntactic structures. It consists of 24 items where the student must read a sentence and select, from four options, the picture that best represents its meaning. It is similar to the Grammatical Structures I test but simpler, requiring the student to only read the sentence and choose the correct image. The reliability coefficient reported in the manual is 0.75.
- Punctuation: This measures the ability to read aloud while respecting punctuation marks. The adolescent must read a text while the examiner records whether they correctly interpret commas, periods, colons, dashes, question marks, and exclamation marks. A total of 30 punctuation marks are scored, reflecting the ability to read fluently with appropriate intonation. The reliability coefficient reported in the manual is 0.77.
- Expository Comprehension: This evaluates the ability to comprehend and retain information from an expository text. Students read the text silently and then answer 10 questions without consulting the text again. The test measures the ability to extract, integrate, and recall information, which is fundamental for learning across subjects. The reliability coefficient reported in the manual is 0.55.
- Narrative Comprehension: Students read a narrative text silently and answer 10 multiple-choice questions, with permission to consult the text. Unlike other tests, the focus is more inferential and there is no time limit. The goal is to measure the ability to extract meaning and build a mental representation of narrative texts, which is essential for understanding and enjoying literature. The reliability coefficient reported in the manual is 0.56.
- Pure Reading Comprehension: The student reads aloud an expository text and then answers 10 questions with the text available, while the total time to complete the test is recorded. The questions are inferential, requiring deep understanding beyond simple recall. It assesses the ability to interpret and analyze expository texts without memory recall influencing performance. The reliability coefficient reported in the manual is 0.57.
- Mnemonic Reading Comprehension: Students read a more complex expository text silently and then answer 10 open-ended questions without consulting the text. This requires greater recall and synthesis effort. The combination of tests with and without access to the text allows analysis of the role of memory in reading comprehension, providing a more comprehensive measure of the ability to process expository texts. The reliability coefficient reported in the manual is 0.67.
- Oral Comprehension: The examiner reads aloud an expository text while the student listens attentively. Then, 10 inferential questions are presented that the student must answer. This test measures comprehension without the influence of reading, as processing is based solely on listening. Comparing oral and reading comprehension performance can help identify potential reading fluency difficulties, such as those associated with dyslexia. The reliability coefficient reported in the manual is 0.64.

Indexes: These are global dimensions that summarize both overall reading ability and each of the processes involved.

- General Reading Index: This index indicates the overall ability to read all types of words, sentences, and texts fluently and accurately and to extract their meaning by integrating it with prior knowledge. The reliability coefficient reported in the manual is 0.94.
- Lexical Index: This index reflects the ability to accurately and quickly recognize familiar and unfamiliar words, as well as to automatically transform and convert letters into sounds. The reliability coefficient reported in the manual is 0.95.
- Syntactic Index: This index reflects the ability to extract the message from sentences and determine the function of words or groups of words by applying different types of rules, as well as reading while respecting pauses and intonation dictated by punctuation marks. The reliability coefficient reported in the manual is 0.91.
- Semantic Index: This index indicates the effectiveness of processes for extracting meaning from texts and forming a mental representation that integrates this message into memory, using both inferential and mnemonic mechanisms. The reliability coefficient reported in the manual is 0.82.

### 2.3. Data Analysis

The statistical analysis was conducted using SPSS version 25 and G*Power version 3.1. The procedure consisted of an initial phase of descriptive analysis, followed by an inferential analysis phase to identify significant relationships and differences among the data.

Subsequently, the normality of the variables was assessed to determine the most appropriate statistical test for each case. Analysis of variance (ANOVA) was then applied to those variables with a normal distribution, aiming to compare means among different groups and identify potential statistically significant differences. For variables that did not meet the normality criterion, the non-parametric Kruskal–Wallis test was used, which is based on data ranks rather than variances, allowing its use when traditional parametric assumptions are not satisfied (McKight & Najab, 2010; Ostertagova et al., 2014).

Additionally, the effect size is reported, providing an estimate of the magnitude of the difference between compared samples, allowing the practical relevance of the observed results to be evaluated beyond statistical significance. For this analysis, Cohen’s d was used, a widely recognized index quantifying effect magnitude, especially effective when working with small to moderate sample sizes (Cohen, 1988). Simultaneously, statistical power (1 − β) was calculated, reflecting the probability of detecting a real effect if it exists, given the sample size and significance level.

Given the number of literacy-related variables analyzed and the relatively small sample size, no formal correction for multiple comparisons (e.g., Bonferroni adjustment) was applied. Such corrections, while reducing the risk of Type I errors, can substantially decrease statistical power and increase the risk of Type II errors, particularly in studies involving rare populations and limited sample sizes (Midway et al., 2020). Therefore, effect sizes and statistical power were considered alongside *p*-values to support a more comprehensive interpretation of the results.

## 3. Results

The analysis of the sample based on sex and group membership reveals a predominance of the male sex, as shown in Table 1. However, using the Chi-square test, no statistically significant differences were observed in sex distribution among the analyzed groups (χ^2^(2) = 0.643; *p* = 0.725), indicating that the proportion of females and males remains similar across gifted non-dyslexic, gifted dyslexic, and non-gifted dyslexic groups. Similarly, the distribution of the sample according to grade level and group membership showed comparable results. Fisher’s Exact Test did not reveal statistically significant differences (*p* = 0.472), suggesting that grade-level distribution is similar among the groups mentioned.

Table 2 and Table 3 outline the primary findings, encompassing statistically significant effects, their corresponding effect size estimates, and statistical power. Table 2 presents descriptive statistics and comparisons across the three groups for processing speed and writing-related measures derived from the PROESC battery, including test type, *p* values, effect sizes, and statistical power. Table 3 provides descriptive statistics and group-based differences for reading-related measures obtained from the PROLEC-SE-R battery. These measures include Indexes, which represent global dimensions summarizing overall reading ability and the processes involved, and Main Scores, which reflect performance and achievement on the tasks used to assess each specific process. Non-significant findings should be interpreted as indicating a lack of statistically significant differences at the predefined alpha level, rather than as evidence of equivalence between groups.

The variable analysis revealed differences between the groups. Regarding processing speed, the G-D group obtained significantly lower scores than the G group (*p* < 0.05) but higher mean scores than the D group, although the latter difference did not reach statistical significance. Thus, the G-D group showed mean processing speed scores between those of the G and D groups.

The results of the writing tests indicate that the G-D group generally showed an intermediate pattern, with mean scores below those of the G group and above those of the D group. However, significant differences between the G-D and G groups were only found in the arbitrary spelling word dictation test (*p* < 0.05). No significant differences were observed between the G-D and G groups in syllable dictation, rule-based word dictation, total pseudoword dictation, pseudoword dictation with spelling rules, story writing, or essay writing. Likewise, no significant differences were found between the G-D and D groups in the writing-related measures analyzed. Therefore, although the G-D group tended to perform between the G and D groups descriptively, statistically significant differences mainly emerged when comparing the G-D group with the G group in arbitrary spelling.

The reading test analysis results show that the G-D group also exhibited an intermediate and heterogeneous pattern. Compared with the G group, the G-D group obtained significantly lower scores in the general reading index (*p* < 0.05), syntactic index (*p* < 0.05), pseudoword reading (*p* < 0.05), and expository comprehension (*p* < 0.05). No significant differences between the G-D and G groups were found in the remaining reading measures. Compared with the D group, the G-D group obtained significantly higher scores in the general reading index (*p* < 0.05) and pure reading comprehension (*p* < 0.01), whereas the remaining differences did not reach statistical significance. Overall, the G-D group differed from the G group mainly in global reading, syntactic processing, pseudoword reading, and expository comprehension, while its advantage over the D group was mainly observed in global reading and pure reading comprehension.

## 4. Discussion

The present study provides empirical evidence of a distinctive literacy profile in twice-exceptional adolescents, characterized by a heterogeneous pattern of performance, with some domains resembling gifted peers and others aligning more closely with dyslexic profiles. When comparing the G-D group with the other two groups, relevant differences were observed across the assessed domains. This was also observed in processing speed, where the G-D group obtained intermediate scores between the G and D groups. Although the difference between the G-D and D groups did not reach statistical significance, this result is consistent with the idea that twice-exceptional adolescents may present cognitive profiles in which strengths associated with giftedness coexist with vulnerabilities in specific cognitive processes. In terms of writing, the G-D group showed a performance pattern consistent with their heterogeneous profile, scoring lower than the G group but higher than the D group. Notably, except for the arbitrary spelling word dictation test, no significant differences were found between the G-D and G groups, whereas significant differences were observed between the G and D groups in most measures. A similar pattern emerged in reading: the G-D group scored between the other two groups on most indicators. However, while differences between G and D were significant in practically all tests, differences between G and G-D were only significant in specific measures, namely the general reading index, syntactic index, pseudoword reading, and expository text comprehension. Additionally, the G-D group differed significantly from the D group in global reading measures, including the general reading index and pure reading comprehension.

These results support the hypothesis that G-D adolescents show relative weaknesses in lexical and syntactic processes, alongside comparatively better performance in semantic skills. Their lower scores in lexical and syntactic indices, in some cases significantly different from the G group, contrast with semantic index scores comparable to those of their gifted peers. This pattern suggests that competencies related to comprehension and meaning may be less affected by dyslexia, possibly due to compensatory mechanisms based on more efficient semantic processing supported by high cognitive ability (van Viersen et al., 2019). In line with the previous results, although the G-D group outperforms the D group in most evaluated variables, statistically significant differences are mainly observed in global measures such as pure reading comprehension and the general reading index, indicating that their advantage is more evident at a general level than in specific subskills.

When comparing these findings with previous research, similar conclusions emerge. Berninger and Abbott (2013) found that students with dyslexia and superior verbal reasoning significantly outperformed those with average verbal reasoning in morphological and syntactic skills, although no differences were observed in phonological and orthographic processing. Likewise, studies by van Viersen et al. (2015, 2016) show that G-D individuals outperform D individuals in most reading and spelling tests, although with scores lower than those of G individuals. These authors conclude that, despite having deficits as severe as those of D individuals, G-D individuals achieve better literacy outcomes due to compensatory abilities. Furthermore, van Viersen et al. (2019) demonstrated that G-D adolescents with resolved dyslexia exhibit stronger linguistic abilities in verbal memory, grammar, and vocabulary than those with persistent dyslexia, although phonological deficits often remain.

When comparing results across studies, it is important to consider methodological and contextual factors that may limit direct comparisons, including differences in group classification criteria, assessment instruments, targeted skills, and participants’ age ranges. In this regard, the present study focuses specifically on secondary school adolescents, whereas previous research has often included younger or more heterogeneous samples. In addition, the linguistic context constitutes a key factor, as the orthographic transparency of the language influences reading and writing development and may affect both the manifestation of dyslexia and the cognitive processes involved in literacy (van Viersen et al., 2017). In transparent orthographies such as Spanish, the regularity of grapheme–phoneme correspondences promotes efficient phonological decoding (Ferreres & López, 2014), which may lead to different literacy profiles than those observed in more opaque languages. Moreover, orthographic transparency has been associated with cross-linguistic variability in both learning mechanisms and prevalence estimates (Bonnetier, 2024). Therefore, the findings of the present study, conducted in Spanish and focused on a relatively underexplored developmental stage, should be interpreted within this linguistic framework, and caution is warranted when generalizing them to languages with different orthographic characteristics.

Framing these findings within established cognitive models of dyslexia, empirical evidence supports both the phonological deficit hypothesis and more integrative approaches such as the multiple-deficit model. Difficulties in phonological processing, including phonological awareness, verbal short-term memory, and rapid automatized naming, are widely recognized as core mechanisms underlying dyslexia, regardless of intellectual level (Snowling et al., 2026; van Viersen et al., 2015). At the same time, the multiple-deficit model proposes that dyslexia results from the interaction of multiple cognitive and environmental risk and protective factors (van Viersen et al., 2015), including processes such as working memory, processing speed, and oral language (Kranz et al., 2024). In twice-exceptional populations, this interaction gives rise to heterogeneous cognitive profiles characterized by the coexistence of strengths and weaknesses, challenging single-deficit explanations (Kranz et al., 2024; Maddocks, 2020). Within this framework, certain cognitive strengths may function as protective factors that support compensatory processes during development (van Viersen et al., 2019).

The findings of this study also highlight the complexity involved in identifying students who are both intellectually gifted and dyslexic, as their profiles do not clearly conform to the typical patterns associated with either condition alone (Nicpon et al., 2011; Snowling & Hulme, 2012; van Viersen et al., 2019). This ambiguity complicates their identification and may limit access to appropriate educational interventions. In this context, the results emphasize the importance of adopting a dual-differentiated educational approach that addresses both strengths and specific learning needs. More specifically, the identification of a differentiated literacy profile in G-D adolescents, characterized by relative weaknesses in lexical and syntactic processes alongside preserved semantic abilities, provides a more nuanced understanding of how dyslexia manifests in this population. This has direct implications for assessment and intervention, as it supports the design of targeted educational strategies that build on strengths while addressing specific difficulties. Understanding the cognitive variables that influence reading and writing processes in this group is essential not only for improving academic outcomes but also for promoting self-esteem and emotional well-being (Bermejo et al., 2013; Valdés et al., 2013). By providing empirical evidence in a linguistic context and developmental stage that have received limited attention, this study contributes to advancing a more precise, context-sensitive, and equitable approach to the identification and support of 2e students.

### Study Limitations and Future Research Directions

Addressing twice-exceptionality in adolescence presents important methodological challenges, particularly due to the difficulty of identifying sufficiently large and clearly defined samples. Given the rarity of this profile, the sample size should be considered when interpreting non-significant findings, which should not be understood as evidence of equivalence between groups. In addition, although all participants in the dyslexic groups had an established diagnosis of dyslexia, the retrospective nature of the study did not allow for a more detailed classification of dyslexia profiles, diagnostic history, or educational support received. Future studies should consider these variables in order to better understand how different developmental and educational trajectories may shape reading and writing performance in twice-exceptional students.

Another relevant consideration concerns the linguistic context. The sample consisted of Spanish-speaking participants, and the transparency of the Spanish orthographic system may have influenced the observed patterns. Consequently, the findings should be interpreted within this linguistic framework, and caution is warranted when generalizing them to languages with more opaque orthographies, such as English.

Future research should include larger and more clearly defined samples, ideally supported by a priori power analyses, as well as longitudinal designs to examine how these students’ profiles evolve over time. From a transdiagnostic perspective, it would also be valuable to examine cognitive indices such as verbal comprehension, fluid or perceptual reasoning, working memory, and processing speed in greater detail, as well as their associations with specific reading and writing outcomes. Combining quantitative approaches with case study designs may further contribute to a more nuanced understanding of individual developmental trajectories and to the design of more precise and context-sensitive educational responses.

## 5. Conclusions

This study provides relevant empirical evidence on the reading and writing profile of adolescents with 2e, specifically those who present both intellectual giftedness and dyslexia (G-D), a population that remains underexplored in the literature, particularly at this developmental stage. By comparing G-D students with gifted peers without dyslexia (G) and students with dyslexia without intellectual giftedness (D), the findings contribute to a more precise characterization of this complex and often underidentified profile.

In relation to the research objectives, the results indicate that G-D adolescents exhibit a distinctive and intermediate performance pattern across literacy-related measures. While they generally performed below the G group and above the D group, these differences were not uniformly reflected across all variables. Specifically, G-D students showed relative weaknesses in lexical and syntactic processes, alongside comparatively stronger performance in semantic skills. Although their advantage over the D group was most clearly reflected in global reading measures, such as the General Reading Index and comprehension tasks, these global indicators should be interpreted together with specific subskill measures, as they may not fully capture the heterogeneous pattern of strengths and weaknesses that characterizes this profile. These findings support the presence of a heterogeneous profile in which cognitive strengths associated with intellectual giftedness may partially compensate for persistent difficulties related to dyslexia. From a broader perspective, the results suggest that the interaction between intellectual giftedness and dyslexia does not simply reflect the additive effects of both conditions but rather gives rise to a unique and heterogeneous literacy profile. This complexity may contribute to the underidentification of these students, as their performance does not fully align with the expected patterns of either giftedness or dyslexia when considered independently. Consequently, the findings highlight the importance of adopting diagnostic, assessment, and educational approaches that are sensitive to this dual profile and that integrate both strengths and difficulties.

Overall, this study contributes to advancing current knowledge by providing empirical evidence within a specific linguistic context, Spanish, characterized by a transparent orthography, and by focusing on adolescence, a developmental stage that has received comparatively little attention in previous research. These findings reinforce the need for more precise identification processes and for the development of educational responses that are better aligned with the specific characteristics of 2e students. Finally, the results open new avenues for future research aimed at further exploring the developmental trajectories of this population, particularly through longitudinal designs that allow for a more nuanced understanding of how these profiles evolve over time. A deeper understanding of these processes will be essential for improving both identification and intervention practices, ultimately contributing to more effective and equitable educational support for students with twice exceptionality. From an individual differences perspective, these results reinforce the need to consider twice-exceptionality as a distinct cognitive profile rather than the simple coexistence of two conditions.

## Figures and Tables

**Table 1:** Descriptive Analysis: Sample Size, Sex Proportion, Grade Level, Age and IQ Score by Group.

				Grade Level						Age			IQ	
Group	n	% Male	% Female	1°	2°	3°	4°	1°Bach	2°Bach	M	SD	Range	M	SD
**Gifted (G)**	46	65.22%	34.78%	16	14	8	4	3	1	13.68	1.41	11.25–16.67	134.20	6.91
**Gifted dyslexic (G-D)**	33	72.73%	27.27%	7	9	6	2	5	4	14.54	1.57	12.17–16.92	132.24	4.61
**Dyslexic (D)**	32	71.88%	28.13%	8	7	9	4	1	3	14.57	1.44	12.42–16.83	112.13	8.26
**Total**	111	69.37%	30.63%	31	30	23	10	9	8	14.19	1.52	11.25–16.92	127.25	11.80

Note. Age is reported in years and was calculated from age in months. M = mean; SD = standard deviation.

**Table 2:** Test Scores and Comparison Results Between Groups: Processing Speed and PROESC Values.

	Gifted (G)		Gifted Dyslexic (G-D)		Dyslexic (D)			G vs. D			G vs. G-D			G-D vs. D		
	M	SD	M	SD	M	SD	T.T	*p*-Value	Effect Size	1 − β	*p*-Value	Effect Size	1 − β	*p*-Value	Effect Size	1 − β
Processing Speed	115.3	13.863	107.39	12.969	101.56	11.208	P	0.001	0.987	0.988	0.025	0.586	0.816	n.s.		
Syllable Dictation	24.05	1.05	23.83	1.197	23.39	1.383	NP	n.s.			n.s.			n.s.		
Word Dictation (arbitrary spelling)	23.85	1.885	22.52	2.798	21.55	3.086	NP	0.001	0.952	0.985	0.042	0.585	0.745	n.s.		
Word Dictation (rule-based spelling)	24.03	1.267	22.97	2.337	21.52	2.827	NP	0.001	1.282	0.999	n.s.			n.s.		
Pseudoword D. (total score)	21.71	1.575	21.17	1.754	20.45	2.03	NP	0.028	0.708	0.881	n.s.			n.s.		
Pseudoword D. (spelling rules)	12.53	1.246	11.93	1.486	11.58	1.689	NP	n.s.			n.s.			n.s.		
Story Writing	6.87	1.576	5.83	1.947	5.71	1.829	NP	0.029	0.686	0.866	n.s.			n.s.		
Essay Writing	6.36	1.693	5.34	2.005	4.9	1.972	NP	0.003	0.804	0.943	n.s.			n.s.		

Note. M = mean; SD = standard deviation; 1 − β = statistical power; T.T. = Test Type; P = parametric; NP = nonparametric; n.s. = non-significant pairwise comparison. Effect size and statistical power are reported for significant pairwise comparisons.

**Table 3:** Test Scores and Comparison Results Between Groups: PROLEC-SE-R Values.

	Gifted (G)		Gifted Dyslexic (G-D)		Dyslexic (D)			G vs. D			G vs. G-D			G-D vs. D		
	M	SD	M	SD	M	SD	T.T	*p*-Value	Effect Size	1 − β	*p*-Value	Effect Size	1 − β	*p*-Value	Effect Size	1 − β
General Reading Index	119.43	10.346	107.95	12.382	95.46	15.452	P	0.001	1.872	0.996	0.04	0.994	0.79	0.026	0.921	0.707
Lexical Index	104.87	13.69	93.4	13.044	82.08	15.354	NP	0.001	1.576	0.989	n.s.			n.s.		
Syntactic Index	125	9.743	111.55	14.573	104.69	15.024	P	0.001	1.653	0.984	0.019	1.069	0.844	n.s.		
Semantic Index	119.14	8.027	116.5	10.817	106.62	13.131	NP	0.021	1.194	0.902	n.s.			n.s.		
Lexical Selection	45.8	3.663	44.38	4.96	42.06	4.249	NP	0.012	0.961	0.909	n.s.			n.s.		
Semantic Categorization	68.72	13.452	58.86	12.952	50.61	14.861	NP	0.001	1.29	0.99	n.s.			n.s.		
Word Reading	159.25	39.266	132.5	36.783	111.46	28.532	P	0.002	1.387	0.947	n.s.			n.s.		
Pseudoword Reading	102.19	23.092	81.75	20.81	72.46	25.142	P	0.003	1.238	0.891	0.031	0.937	0.774	n.s.		
Grammatical Structures I	22.56	1.758	20.55	3.439	18.22	3.246	NP	0.001	1.823	0.999	n.s.			n.s.		
Grammaticality Judgments	22.64	5.476	18.79	6.472	17.39	6.98	P	0.026	0.86	0.775	n.s.			n.s.		
Grammatical Structures II	23.13	1.06	21.75	2.221	20.92	2.139	NP	0.006	1.416	0.971	n.s.			n.s.		
Punctuation	30	0	29.9	0.447	30	0	NP	n.s.			n.s.			n.s.		
Expository Comprehension	9.52	0.714	8.62	1.449	8.78	1.003	NP	0.04	0.886	0.866	0.026	0.812	0.888	n.s.		
Narrative Comprehension	7	1.555	6.93	1.926	6.72	1.841	NP	n.s.			n.s.			n.s.		
Pure Reading Comprehension	8.94	0.929	8.65	1.531	7.31	1.377	NP	0.006	1.442	0.978	n.s.			0.009	0.912	0.787
Mnemonic Reading Comprehension	8.93	0.961	8.5	1.469	7.38	1.502	NP	0.025	1.279	0.94	n.s.			n.s.		
Oral Comprehension	8.87	0.99	8.2	1.197	7.46	1.506	NP	0.022	1.146	0.89	n.s.			n.s.		

Note. M = mean; SD = standard deviation; 1 − β = statistical power; T.T. = Test Type; P = parametric; NP = nonparametric; n.s. = non-significant pairwise comparison. Effect size and statistical power are reported for significant pairwise comparisons.

## Data Availability

The original contributions presented in this study are included in the article. Further inquiries can be directed to the corresponding author.
